# Single pairing spike-timing dependent plasticity in BiFeO_3_ memristors with a time window of 25 ms to 125 μs

**DOI:** 10.3389/fnins.2015.00227

**Published:** 2015-06-30

**Authors:** Nan Du, Mahdi Kiani, Christian G. Mayr, Tiangui You, Danilo Bürger, Ilona Skorupa, Oliver G. Schmidt, Heidemarie Schmidt

**Affiliations:** ^1^Material Systems for Nanoelectronics, Faculty of Electrical and Information Engineering, Chemnitz University of TechnologyChemnitz, Germany; ^2^Neuromorphic Cognitive Systems Group, Institute of Neuroinformatics, University of Zurich and ETH ZurichZurich, Switzerland; ^3^Semiconductor Materials, Institute of Ion Beam Physics and Materials Research, HZDR Innovation GmbHDresden, Germany; ^4^Institute for Integrative Nanosciences, IFW DresdenDresden, Germany

**Keywords:** BiFeO_3_ memristor, artificial synapse, single pairing STDP, memory consolidation, learning window, low-power device

## Abstract

Memristive devices are popular among neuromorphic engineers for their ability to emulate forms of spike-driven synaptic plasticity by applying specific voltage and current waveforms at their two terminals. In this paper, we investigate spike-timing dependent plasticity (STDP) with a single pairing of one presynaptic voltage spike and one post-synaptic voltage spike in a BiFeO_3_ memristive device. In most memristive materials the learning window is primarily a function of the material characteristics and not of the applied waveform. In contrast, we show that the analog resistive switching of the developed artificial synapses allows to adjust the learning time constant of the STDP function from 25 ms to 125 μs via the duration of applied voltage spikes. Also, as the induced weight change may degrade, we investigate the remanence of the resistance change for several hours after analog resistive switching, thus emulating the processes expected in biological synapses. As the power consumption is a major constraint in neuromorphic circuits, we show methods to reduce the consumed energy per setting pulse to only 4.5 pJ in the developed artificial synapses.

## Introduction

Since the discovery of spike-timing dependent plasticity (STDP) in biological synapses (Bi and Poo, 1998; Snider, 2008; Di Lorenzo and Victor, 2013), scientists have been captivated by the idea of changing the synaptic weight, i.e., the strength between the pre- and post-neuron, in bioinspired electronic systems in a fashion similar to biology (Indiveri et al., 2006). However, the circuit-oriented approach is complicated because the “synaptic weight” variable has to be stored typically either as charge in a capacitor (Koickal et al., 2006) or even digitally in neuromorphic IC (Schemmel et al., 2012; Mayr et al., 2013). This adds circuit complexity and increases energy consumption (Indiveri et al., 2006; Adee, 2009; Ananthanarayanan et al., 2009). Therefore, nonvolatile analog resistive switches, namely resistive random-access memory (RRAM) or memristors (Chua, 1971; Du et al., 2013), responding to well-defined input signals by suitably changing their internal state (“weight”) are currently developed. For example, the emulation of STDP with 60–80 pairings of pre- and post-synaptic spikes has been shown for artificial synapses based on memristive TiO_x_ (Seo et al., 2011; Thomas and Kaltschmidt, 2014), WO_x_ (Chang et al., 2011), HfO_x_ (Yu et al., 2011), GST (Kuzum et al., 2012), and on the memristive BiFeO_3_ (Mayr et al., 2012; Cederström et al., 2013).

**Figure 1:**
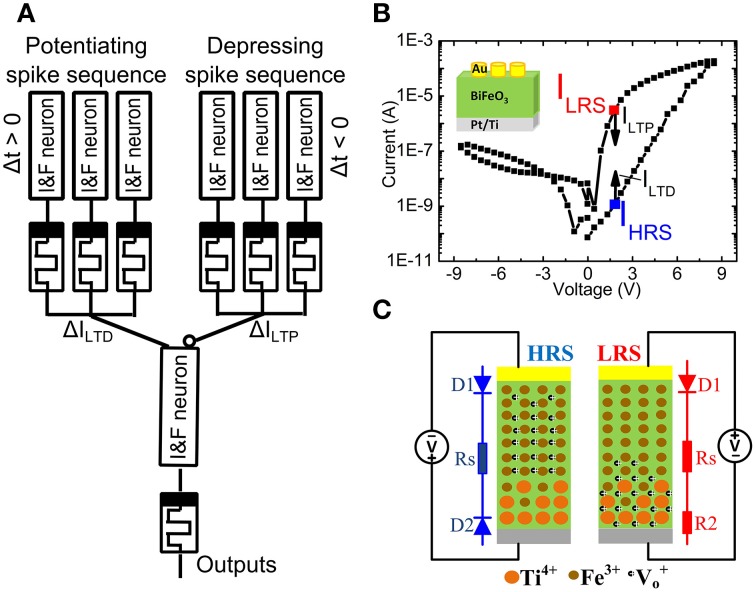
**(A)** Schematic illustration of the memristor-based synaptic electronics. The artificial synapses are placed between Integrate & Fire neurons (I&F neuron). With a well-defined time delay Δt between the pre- and post-spikes the internal state (“weight”) of the memristor is suitably changed. **(B)** Hysteretic current-voltage (IV) characteristics of a Au/BiFeO_3_/Pt memristor in LRS and HRS with a top electrode area of 4.5E4 μm^2^ under source voltages with maximum sweeping pulse amplitude of 8.5 V and a pulse width of 100 ms. The current in high resistance state I_HRS_ and in low resistance state I_LRS_ is read out at +2.0 V, after having switched the memristor into HRS and LRS, respectively. The long term potentiation current I_LTP_ and the long term depression current I_LTD_ lie below the reading current in LRS (I_LRS_) and HRS (I_HRS_). Inset shows the structure of a BFO memristor. **(C)** Schematic demonstration of the distribution of fixed Ti^4+^, fixed Fe^3+^ and mobile V^+^_*o*_.

Figure 1A shows a memristor between the electrical Integrate & Fire (I&F) neurons. The synaptic weight of the memristor can be controlled by the time delay Δt between pre- and post-spike from the 1st layer I&F neuron (Figure 1A) (Zamarreño-Ramos et al., 2011). The 2nd layer I&F neuron sums up the signals from all incoming neurons and generates voltage spikes transmitted to other neurons (not shown) through memristor-based artificial synapses. The memristive BiFeO_3_(BFO) can serve as an analog resistive switch (Shuai et al., 2011) with multiple distinguishable low resistance states (LRSs) (Shuai et al., 2013; Jin et al., 2014) and with a single detectable high resistance state (HRS). Due to the thermal diffusion of Ti atoms and their substitutional incorporation into the lower part of the BiFeO_3_ (BFO) layer during BFO thin film growth on a Pt/Ti bottom electrode, the barrier at the Pt/Ti bottom electrode is flexible.

Earlier we have shown that STDP and triplet plasticity with learning windows on the millisecond time scale can be faithfully emulated on BFO-based artificial synapses by applying 60–80 pairings of pre- and post-synaptic spikes (Mayr et al., 2012; Cederström et al., 2013). In this work we investigate a significantly wider range of timescale configurability, ranging from 25 ms to 125 μs. To the best of our knowledge, this kind of timescale configurability has not been shown in memristive synapses before. We also examine the evolution of the induced memristive weight change over time and provide several power consumption figures. By increasing the programming voltage (HRS/LRS writing pulse amplitude), it is possible to decrease the switching pulse width as well as the power consumption during a single STDP writing process on BFO-based artificial synapses. Furthermore, the increased programming voltage also shortens the total pairing spike time, and enables to move from the standard biology-like 60–80 spike pairing STDP experiment to a single pairing STDP experiment that results in the same weight/memristance change.

Our work is structured as follows: In Section Materials and Methods, we describe the non-volatile resistive switching of BFO–based artificial synapses and introduce the single pairing STDP pulse sequence. In Section Results, we present the measured learning window, memory consolidation, and energy consumption of the single pairing STDP in BFO-based artificial synapses and discuss configurability, energy consumption, and retention of weight change in Section Discussion. The paper is summarized and an outlook is given in Section Summary and Outlook.

## Materials and methods

### Nonvolatile, analog resistive switching in BiFeO_3_

Polycrystalline, 600 nm thick BiFeO_3_ (BFO) thin films with a flexible bottom barrier have been grown by pulsed laser deposition on Pt/Ti/SiO_2_/Si substrates. Circular Au top contacts have been magnetron sputtered on the BFO thin films using a shadow mask (Shuai et al., 2011, 2013; Jin et al., 2014). The Pt/Ti bottom electrode and the Au top contacts posses a flexible and a fixed barrier height, respectively. As illustrated in Figure 1B, by applying the sweeping source voltage from 0 V → −8.5 V → +8.5 V → 0 V between the Au top electrode and the bottom electrode, the current-voltage characteristics, which were recorded using a Keithley source meter 2400, reveal reproducible nonvolatile hysteretic bipolar resistive switching in BFO memristors with mobile donors (oxygen vacancies) and fixed donors (Ti donors). As illustrated in Figure 1C which has been adapted from Ref. (You et al., 2014), the physical mechanism underlying resistive switching in BFO memristors is related with the nonvolatile change of flexible barriers in Ti-containing BFO memristors. Due to voltage application of a LRS writing pulse, fixed Ti donors close to the bottom electrode can effectively trap mobile oxygen vacancies in BFO. The bottom electrode becomes non-rectifying and the BFO memristor is in LRS. On the other hand, when applying the HRS switching pulse, the mobile donors in BFO memristors are redistributed between the top and the bottom electrode. The bottom electrode becomes rectifying and the BFO memristor is in HRS. Note that for both writing pulses the Au top electrode remains rectifying.

A single writing pulse with an amplitude *V_w_* = +8.0 V and −8.0 V can be used to switch the BFO memristor into LRS and HRS, respectively. The maximum possible amplitude increases with the thickness of the BFO memristor and decreases with the length of the writing pulse. For a BFO layer thickness of 600 nm and a writing pulse length of 100 ms, the barrier height of the bottom electrode typically starts to change at a writing pulse of amplitude *V_w_* = +3.0 V. Applying a dc voltage below +2.0 V to the BFO memristor does not change the barrier height of the bottom electrode, and the state of the BFO memristor does not change. Therefore, the +2.0 V dc voltage is defined as the reading bias for the 600 nm thick BFO memristor. The ratio between the resistance R_HRS_ in HRS and the resistance R_LRS_ in LRS amounts to R_HRS_/R_LRS_ = 2770 (Figure 1B). For changing the synaptic weight the absolute value of the amplitude V_p_ of the pre-synaptic and post-synaptic spike has to be larger than the reading bias amplitude +2.0 V (Smerieri et al., 2008; Borghetti et al., 2009; Lai et al., 2009). In our previous work, we used a 500 nm thick BFO layer and an amplitude of 2.3 and 2.0 V for STDP with 60–80 pairings of pre- and post-synaptic spikes. In this work, we use a 600 nm thick BFO layer and an amplitude V_p_ of 3.0 V for STDP with single pairing of pre- and post-synaptic spikes. For the potentiating (depressing) spike sequence, the long term potentiation current I_LTP_ (long-term depression current I_LTD_) decreases exponentially with decreased pulse amplitude in positive (negative) voltage range: I_LRS_ > I_LTP_ (I_HRS_ < I_LTD_).

**Figure 2:**
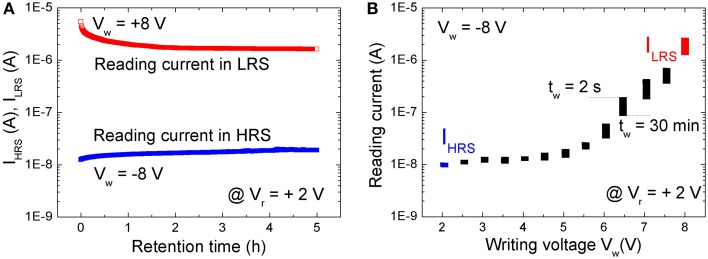
**(A)** Retention test with a reading bias of *V_r_* = +2.0 V after setting the BFO memristor to LRS (red symbols) and to HRS (blue symbols). The reading current has been recorded every 30 s. **(B)** Retention of multilevel resistive switching in a BFO memristor, which has been initially set to HRS by a writing voltage of *V_w_* = −8.0 V. The reading current has been measured at a small reading bias of *V_r_* = +2.0 V directly after switching BFO into one of the multiple LRSs with a positive writing bias of V_w_ ranging from +2.0 to +8.0 V (top edge of the rectangles, *t_w_* = 2 s) and 30 min later (bottom edge of the rectangles, *t_w_* = 30 min). Note that the reading current starts to increase for a writing voltage of ca. +3.0 V, i.e., the state of the BFO starts to change. All states in **(B)** are read with a pulsed reading bias amplitude of *V_r_* = +2.0 V and length 100 ms. Because the reading current changes from *I_r_* = 1.1E-2 μA in HRS with *R* = 1.8E8 Ω to *I_r_* = 2 μA in LRS with *R* = 1E6 Ω, the power (*P* = *R* · *I*^2^) will change from 2.2E-8 W in HRS to 4.0E-6 W in LRS. The resolution of a pulsed power meter amounts to 0.01 dB. So theoretically more than 2000 power levels would be achievable, and we expect that at least 32/64 levels are possible in a power efficient manner.

The nonvolatile resistive switching of BFO was examined by a retention test (Figure 2A). A single writing pulse of *V_w_* = +8.0 V and − 8.0 V and a pulse width of *t_p_* = 100 ms was used to switch the BFO memristor into LRS and HRS, respectively. The reading currents have been read out with a reading bias of *V_r_* = +2.0 V and are defined as the current of HRS (I_HRS_) and LRS (I_LRS_). As shown in Figure 2A the BFO memristor exhibits degradation of the LRS within the testing time of 2 h. No significant change has been observed for HRS during the retention time of 5 h. This non-ideal retention motivated us to investigate memory consolidation (Clopath et al., 2008) in BFO with the shortened pulse sequence of single pairing STDP.

A BFO memristor with multilevel resistive switching can be considered as an analog resistive switch and used as an artificial synapses. The retention of multilevel resistive switching is illustrated in Figure 2B. Positive writing pulses ranging from 2.0 to 8.0 V are applied to the BFO-based artificial synapse. As expected from the current-voltage characteristics (Figure 1B), the reading current at 2.0 V increases with increasing amplitude of the writing bias. After applying the positive writing pulses *V_w_* (as switched, *t_w_* = 2 s), the reading current was largest and slightly decreased (30 mins, *t_w_* = 30 min) with increasing waiting time t_*w*_ (Figure 2B). However, due to the degradation (Figure 2B) different LRSs will become indistinguishable. E.g., the reading current for a writing bias of *V_w_* = 5.5 V and a waiting time of *t_w_* = 2 s is the same as the reading current for *V_w_* = 6.0 V and *t_w_* = 30 min. We have already shown that the retention of BFO memristors can be significantly improved by an additional BFO surface modification using low energy Ar^+^ ion irradiation before depositing the Au top electrode (Shuai et al., 2011). Optimized parameters for the Ar^+^ irradiation process are discussed in Ref. (Ou et al., 2013). The Ar^+^ irradiation helps to homogenize the average crystallite size in the polycrystalline BFO memristors.

### Pulse sequence for single pairing spike-timing dependent plasticity

**Figure 3:**
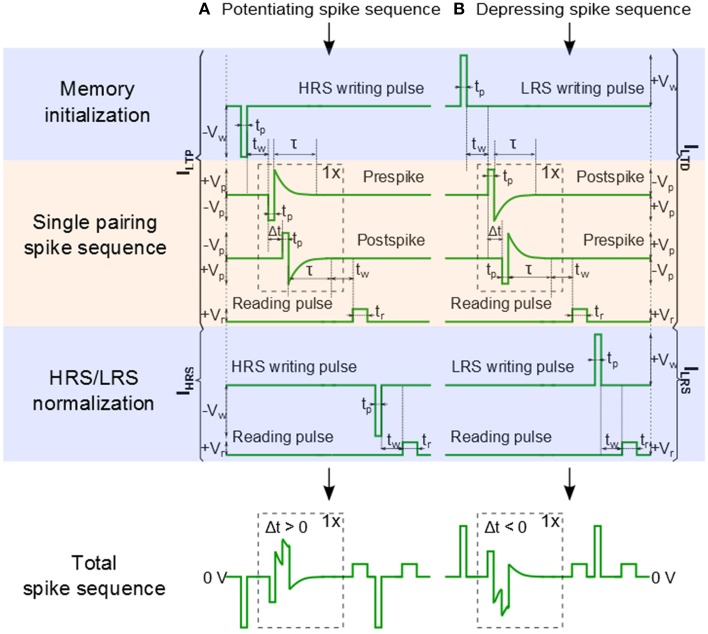
**Signal scheme of Memristor initialization, Single pairing STDP, and Memory consolidation. (A)** A pre-post spike order is used for long term potentiation (LTP). **(B)** A post-pre spike order is used for long term depression (LTD). The potentiation current I_LTP_ (depression current I_LTD_)and the initial HRS current I_HRS_ (and the initial LRS current I_LRS_) are used to normalize the long term potentiation current ΔI_LTP_ (the long term depression current ΔI_LTD_) as defined in Equations (2) and (3). t_*p*_ is the pulse width and t_*w*_ is the measurement waiting time before applying the reading pulse V_*r*_.

In our previous work, we have used a bias amplitude of *V_p_* = 2.3 V for STDP with 60–80 pairings of pre- and post-synaptic spikes (Mayr et al., 2012; Cederström et al., 2013). Especially, Mayr et al. illustrates how the pre- and post-synaptic waveforms of a specific biology-derived synaptic plasticity rule (Mayr and Partzsch, 2010) can be adjusted to operate the BFO memristors. The resulting waveforms are comparable to the waveforms proposed by Zamarreño-Ramos et al. (2011). In order to shorten the total pairing spike time, in this work we slightly increased the bias amplitude to *V_p_* = 3.0 V and applied a single pre- and post-synaptic spike. In comparison to what is discussed in Mayr et al. (2012), the single spike pairing instead of multiple (60–80) pairings allows us to shorten the total spike time and to adjust the learning time constant of the STDP function from 25 ms to 125 μs. The detailed signal scheme of Memristor initialization, single pairing STDP, and memory consolidation for long-term potentiation (LTP) and long-term depression (LTD) are shown in Figure 3. In order to facilitate reproducing this signal scheme, the parameters used in every step in the pulse sequence are listed in Table 1. As illustrated in **Figure 6A** the signal scheme for resistive switching from HRS into a single LRS (**Figure 6B**) can be simplified and reduced to Memristor initialization for LTP and to Memory consolidation for LTD (**Figure 6A**). The step labeled Memristor initialization refers to the application of a writing pulse to set the BFO memristor in HRS and LRS. In the HRS the BFO memristor has both rectifying top and bottom electrodes whereas in the LRS the BFO memristor has a rectifying top electrode and a non-rectifying bottom electrode (You et al., 2014). For the pulse order leading to potentiation (Figure 3A), a single negative pulse, i.e., the HRS writing pulse, is applied to switch the memristive device into HRS. After the waiting time t_*w*_ a single pre- and a single post-spike is applied to the top electrode of device. The pre- and post-spikes superimpose at the BFO memristor as potentiating spike, and the spike timing difference Δt determines the waveform of the potentiating spike (Δt = *t_p_* > 0 for the potentiating inputs). Each pre- and post-spike consists of one rectangular pulse with pulse amplitude V_*p*_ and one exponentially decaying pulse V_exp_

(1) $$V_{exp}=|V_{p}|•exp\left(\frac{-t}{\tau }\right),$$

with the decay time τ = τ_pre_ = τ_post_, where τ_pre_ and τ_post_ are the exponential decay times of pre- and post-spikes, respectively. In order to reduce the influence of the exponential decay on the single pairing STDP function, we choose τ = 2.5 · *t_p_*. For the potentiating (depressing) spike order, the spike timing difference Δt between the pre- and post-spike is positive (negative) and lies in the range: *t_p_* = |Δ*t*| = 10 · *t_p_*. In both pre- and post-spikes, the rectangular pulse is short compared to the decay time of the exponential waveform, and the amplitude of the overlapped spike pulses depends on the spike time difference Δt between both waveforms. After the measurement waiting time *t_w_* the synaptic weight of BFO-based artificial synapses has been checked by applying a reading bias of *V_r_* = +2.0 V with a pulse width of *t_r_* = 100 ms. The reading current is defined as the potentiation current I_LTP_ anddepression current I_LTD_ after sourcing potentiating spike and depressing spike, respectively.

**Table 1:** **Parameters for the potentiating spike sequence (Δt > 0) and for the depressing spike sequence (Δt < 0) during Memristor initialization, Memory consolidation, and Single pairing STDP**.

**Step in pulse sequence**	**Memristor initialization**	**Memory consolidation**	**Single pairing STDP**	**Memory consolidation**	**Memory consolidation**
Potentiating spike sequence	−*V_w_* & *t_p_*	*t_w_*	−*V_p_* & *t_p_*/+*V_p_* & τΔ*t* > 0 +*V_p_* & *t_p_*/−*V_p_* & τ	*t_w_*	+*V_r_* & *t_r_*
Depressing spike sequence	+*V_w_* & *t_p_*	*t_w_*	+*V_p_* & *t_p_*/−*V_p_* & τΔ*t* < 0 −*V_p_* & *t_p_*/+*V_p_* & τ	*t_w_*	+*V_r_* & *t_r_*

*The amplitude |V_w_| and the length t_p_ of the writing bias pulse determine the Memristor initialization. The waiting time t_w_ after Memristor initialization, the waiting time t_w_ after Single pairing STDP and the amplitude |V_r_| and the length t_r_ of the reading bias pulse determine Memory consolidation. The amplitude −V_p_, the length t_p_, the amplitude +V_p_ and exponential decay τ determine the presynaptic spike and the amplitude +V_p_, the length t_p_, the amplitude −V_p_, and the exponential decay τ determine the post-synaptic spike. The time delay between the pre- and the post-spike is defined by Δt*.

Finally, the reading current I_HRS_ (I_LRS_) of BFO in HRS (LRS) is measured at a reading bias of *V_r_* = +2.0 V after recording I_LTP_ (I_LTD_). For biological reasons it is desirable to keep STDP bounded. Therefore, we have normalized the LTP and LTD current values. After a potentiating spike sequence the synaptic weight scales with the normalized potentiation current ΔI_LTP_

(2) $$\Delta I_{LTP}\left(\%\right)=\frac{I_{LTP}-I_{HRS}}{I_{LTP}}*100\%,$$

and after a depressing spike sequence the synaptic weight scales with the normalized depression current ΔI_LTD_

(3) $$\Delta I_{LTD}\left(\%\right)=\frac{I_{LTD}-I_{LRS}}{I_{LRS}}*100\%.$$

After normalization using Equations (2) and (3) LTP lies in the range from 0 to +100% and LTD lies in the range from 0 to −100%, respectively. As we have shown in Mayr et al. (2012), the specific STDP characteristics can be configured through the waveform. Specifically, τ_pre_ directly translates to the STDP pre-post time window, while τ_post_ translates to the post-pre time window. The *V_p_* of the pre- and post-pulses translate to the respective scaling of the STDP amplitudes.

## Results

In the following single pairing STDP in BFO-based artificial synapses (Section Nonvolatile, Analog Resistive Switching in BiFeO3) is demonstrated by using different pulse widths *t_p_* and measurement waiting times *t_w_*. The potentiating and depressing input signals (Section Pulse Sequence for Single Pairing Spike-timing Dependent Plasticity) have been generated with an Agilent pulse function arbitrary generator 81150A. The reading current has been measured with a Keithley 2400 source meter.

### Learning window

**Figure 4:**
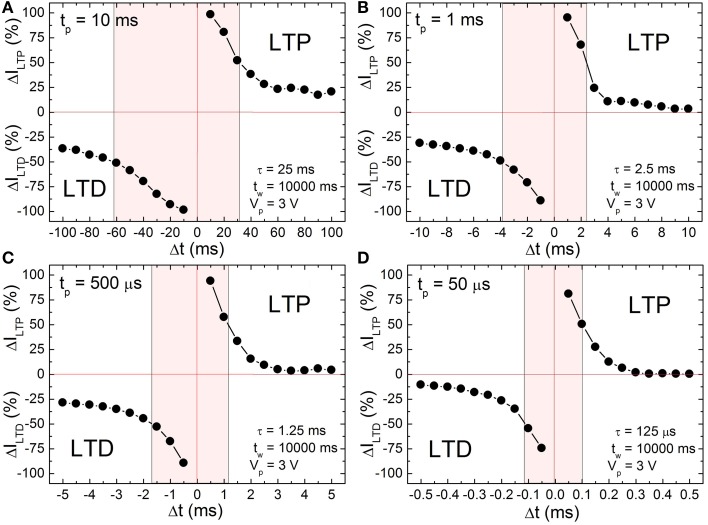
**Long term depression current ΔI_LTD_ (negative range of y-axis) and long term potentiation current ΔI_LTP_ (positive range of y-axis) of a ca. 600 nm thick BFO memristor with a contact area of 4.5E4 μm^2^ for single pairing STDP with pulse width (A)** ***t_p_*** **= 10 ms, (B)** ***t_p_*** **= 1 ms, (C)** ***t_p_*** **= 500 μs and (D)** ***t_p_*** **= 50 μs, measurement waiting time** ***t_w_*** **= 10000 ms, pulse amplitude** ***V_p_*** **= 3.0 V, reading pulse amplitude** ***V_r_*** **= +2.0 V and reading pulse width** ***t_r_*** **= 100 ms**. ΔI_LTD_ and ΔI_LTP_ have been normalized using Equations (2) and (3), respectively. The memristor was preset in HRS and LRS (Memristor initialization in Table 1) with a writing pulse amplitude of *V_w_* = −8.0 V and *V_w_* = +8.0 V, respectively.

According to the input signal scheme (Figure 3) the BFO memristor is set in the HRS and in the LRS with a writing pulse amplitude of *V_w_* = −8.0 and +8.0 V, respectively. For the single pairing STDP measurements on a BFO-based artificial synapse pre- and post-spikes of different pulse widths *t_p_* = 10 ms, 1 ms, 500 μs, and 50 μs, and with a pulse amplitude of |±*V_p_*| = 3.0 V, and a waiting time *t_w_* 10 s have been chosen (Figure 4). The exponential decay time constant (τ = 2.5 · *t_p_*) amounts to τ = 25 ms (Figure 4A), 2.5 ms (Figure 4B), 1.25 ms (Figure 4C), and 125 μs (Figure 4D). After recording I_LTP_ (I_LTD_) the reading current I_HRS_ (I_LRS_) of BFO in HRS (LRS) has been measured at a reading bias of *V_r_* = +2.0 V and the normalized potentiation current ΔI_LTP_ Equation (2) and the normalized depression current ΔI_LTD_ Equation (3) are calculated. The synaptic weight of the BFO memristor scales with the normalized potentiation current ΔI_LTP_ and the normalized depression current ΔI_LTD_. If the prespike precedes the post-spike (Δt > 0) biological synapses (Bi and Poo, 1998) undergo long term potentiation LTP, i.e., the connection between two neurons becomes stronger. On the other hand, if the post-spike precedes the prespike (Δt < 0), biological synapses undergo long term depression LTD, i.e., the connection between two neurons becomes weaker. We have measured the LTD current I_LTD_ and the LTP current I_LTP_ in a BFO-based artificial synapse and can show that the BFO memristor emulates the STDP function of biological synapses. The normalized current ΔI decreases with increasing delay time |Δt|. The normalized current curve for positive and negative Δt is the LTP and LTD curve (Figure 4), respectively. As an example, in the following we discuss the LTP curve in Figure 4 for Δt = *t_p_* > 0. Initially the BFO-based artificial synapse is set into HRS. The maximum amplitude of the potentiating spike amounts to 2*V_p_* = +6.0 V. For this potentiating spike the BFO-based artificial synapse is fully switched to LRS. The normalized potentiation current ΔI_LTP_ at Δ*t* = *t_p_* amounts to ca. 100%. In the time delay range 0 < *t_p_* < Δ*t* ≤ 10 · *t_p_*, the maximum amplitude of potentiating spikes is reduced from 6.0 to 3.2 V. Therefore, the exponential-like decay of the normalized current dominates STDP with increasing Δt and the synapse cannot be fully switched to LRS by applying these potentiating spikes. For both positive and negative time delays |Δ*t*| = 10 · *t_p_*, ΔI decreases with decreasing pulse width *t_p_*. At *t_p_* = 500 μs and 50 μs, ΔI_LTP_ amounts to 0% at |Δ*t*| = 10 · *t_p_*. It is also noticed that ΔI_LTP_ decreases more strongly than ΔI_LTD_ in the larger time delay range. That is because the threshold voltage for LRS is higher than the threshold voltage for HRS. For example in Ref. (Mayr et al., 2012) a voltage of 2.3 V and of 2.0 V has been used as the threshold voltage to switch a BFO-based artificial synapse to LRS and HRS, respectively. The shaded regions in Figure 4 show the ranges of the delay time Δt where the normalized current is larger than 50% for four different pulse widths *t_p_*. This range is also called learning window and decreases from 25 ms to 125 μs with decreasing pulse width *t_p_* from 10 ms to 50 μs.

As can be seen from Figure 4, the STDP time windows can be finely controlled. Specifically, making Δt longer results in a monotonous decrease in both potentiation and depression with increasing Δt, i.e., the memristance change directly and fine grainedly follows the applied waveform resulting from the overlay of pre- and post-pulse. This is in contrast to most other reported memristive synapses, where the time difference between pre- and post-pulse only translates to a stochastic, average change of memristance (Jo et al., 2010; Alibart et al., 2012).

### Memory consolidation

**Figure 5:**
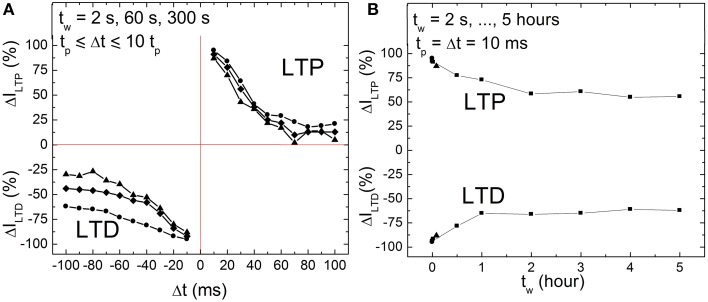
**(A)** STDP of a BFO-based artificial synapses with different waiting times *t_w_* = 2 s (circles), 1 min (quadrangles), and 5 min (triangles) for *t_p_* = Δ*t* = 10 *t_p_*. Pulse amplitude *V_p_* = 3.0 V, pulse width *t_p_* = 10 ms, and exponential decay time τ = 25 ms. **(B)** Memristance weight consolidation for a fixed Δ*t* = *t_p_* = 10 ms and for a waiting time of *t_w_* = 2 s (circles), 60 s (quadrangles), and 300 s (triangles) from **(A)** and *t_w_* = 0.5, 1, 2, 3, 4, 5 h (squares). The pulse amplitude *V_p_* amounts to 3.0 V. The exponential decay amounts to τ = 25 ms. The writing voltage for Memristor initialization amounts to |±*V_W_*| = 6.0 V.

Memory consolidation has been investigated in models of biology in order to improve the understanding of the translation of an initially induced weight change to long term weight stabilization (Anokhin, 2005; Clopath et al., 2008). This motivated us to investigate the memristance weight, i.e., memory consolidation, in BFO-based artificial synapses in more details by performing single pairing STDP measurements with different waiting times *t_w_* (2 s = *t_w_* = 5 h). In biological systems, the waiting time corresponds to the time which elapses before something learned is retrieved. On the other hand, for the memory consolidation measurements, we have again used the ca. 600 nm thick BFO-based artificial synapses and applied a writing voltage of *V_w_* = +6.0 V. In Figure 5A the corresponding STDP data are plotted for *t_w_* = 2, 60, and 300 s. We have chosen single pre- and post-synaptic spikes with the same absolute value of the pulse amplitude *V_p_* = 3.0 V, pulse width *t_p_* = 10 ms and exponential decay time τ = 25 ms. As shown in Figure 5A, the LTP and LTD curves shift toward low normalized current values with increasing waiting time in both positive and negative spike timing ranges. Therefore, the dependence of LTP and LTD on the writing pulse amplitude can be used to trace differences in the LTP and LTD curves of single pairing STDP. For BFO-based artificial synapses with a smaller writing voltage *V_w_*, the optimized STDP curve with more significant exponential-like function (as shown in Figure 4) is reproducible by choosing a smaller pulse amplitude V_*p*_, e.g., *V_p_* = 2.5 V.

Furthermore, memory consolidation measurements (Figure 5B) reveal that for a waiting time t_*w*_ shorter than 1 h there is a visible change of reading current (degradation) both in positive and negative spike timing ranges after applying a single pre-synaptic and post-synaptic pulse sequence, whereas for a waiting time *t_w_* longer than 2 h the current is stabilized. This is in agreement with the results from retention measurements (Figure 2A).

### Energy consumption

Low energy efficiency, large chip size, and complex STDP synapse circuits are major bottlenecks of today's bio-inspired systems, e.g., neural networks where synapses typically outnumber neurons by more than 500:1. In order to reliably observe STDP functionality the corresponding current changes should lie in the nA current range and above. In addition to the stabilization of multilevel resistive switching, we can also increase the current level in a controlled manner by low-energy Ar^+^ ion irradiation (Ou et al., 2013). This will allow for integrating BFO-based artificial synapses with smaller contact area A (Table 2), e.g., in neural networks, without adding another device for amplifying current changes. The estimated energy consumption of each synapse in human brain amounts to only 1–10 fJ (Table 2). In order to approach the high energy efficiency of biological synapses, we applied single pairing (not 60–80 pairing) STDP pulses to BFO-based artificial synapses. For single pairing STDP most of the energy is consumed during SET operation, e.g., Memristor initialization into LRS (Table 1, Figure 3). For example, in TiN/Ge_2_Sb_2_Te_5_/TiN/W artificial synapses the energy for SET operation is 50 pJ while the energy for RESET operation is 0.675 pJ Ref. (Kuzum et al., 2012).

**Table 2:** **Energy consumption E, setting potential amplitude** ***V_w_***, **average setting current I_avg_, pulse width** ***t_p_*** **and top electrode area size A of resistive switching during SET operation of different memristor-based artificial synapses (Kandel and Schwartz, 1985; Jo et al., 2010; Chang et al., 2011; Yu et al., 2011; Kuzum et al., 2012; Wu et al., 2012)**.

**Single synapse**	**E (pJ)**	***V_w_* (*V*)**	**I_avg_ (μA)**	***t_p_* (ns)**	**A (μm^2^)**
Human brain (total number of synapses *N* = 10 ^15^, *P_total_* = 10 W) (Kandel and Schwartz, 1985; Da Costa, 2013)	(1–10) ^*^1E–3	−	−	−	0.12
TiN/Ti/AlO_*x*_/TiN/Ti (Wu et al., 2012)	1.5	+1.5	+100	10	0.72
Au/BFO/Pt/Ti (this paper)	4.7	+23.0	+4.1	50	4.5E+4
TiN/HfO_*x*_/AlO_*x*_/Pt (Yu et al., 2011)	6.0	−2.5	−240	10	0.0079
TiN/Ge_2_Sb_2_Te_5_/TiN/W (Kuzum et al., 2012)	50	−5.5	−900	10	0.018
CMOS-electrode/Ag + Si/CMOS-electrode (Jo and Lu, 2008)	430	+3.2	+0.45	3.0E+5	0.031
Pd/WO_*x*_/W/SiO_2_/Si (Chang et al., 2011)	520	+1.3	+0.40	1.0E+6	0.053

**Figure 6:**
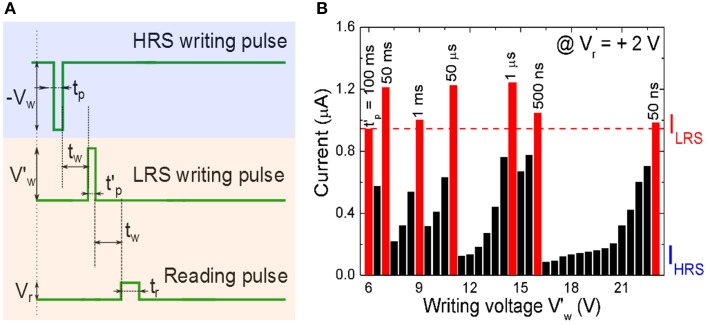
**(A)** Signal scheme for resistive switching a BFO memristor in HRS into LRS. The memristor is initialized into the HRS by applying a writing voltage *V_w_* = −6.0 V with a pulse width *t_p_* = 100 ms, and is then switched back to different LRSs with different pulse amplitudes *V*′_*w*_ and pulse widths *t*′_*p*_. **(B)** Reading current of the BFO memristor with a contact area of 4.5E4 μm^2^ in LRS in dependence on the writing voltage *V*′_*w*_ in the range from 6.0 to 23.0 V and with different constant pulse widths of *t*′_*p*_ = 50 ms, 1 ms, 50 μs, 1 μs, 500 ns, and 50 ns. The reading voltage amounts to +2.0 V. For a given pulse width at least one writing voltage (red bar) is large enough to set the BFO memristor in the LRS. In that case the reading currents is even larger than the current I_LRS_ read out after applying a writing voltage of *V_w_* = +6.0 V with a pulse width of *t*′_*p*_ = 100 ms (first red bar).

The energy consumed during SET operation is

(4) $$E\;=\;{V^{\prime }}_{w}•\;I_{avg}•\;{t^{\prime }}_{p},$$

with *I_avg_* = *I_peak_*/2. The writing voltage amplitude *V_w_*, the setting current *I_peak_*, and writing pulse width *t_p_* are the crucial parameters for evaluating the energy consumption. Note that for the polycrystalline BFO memristors with different sizes of BFO crystallites, larger BFO crystallites below the top electrode are possibly not switchable. Therefore, the effective area of the top electrode might be smaller than the nominal area of the top electrode. Using BFO-based artificial synapses we can downscale the size of the top electrodes (Jin et al., 2014), increase the pulse amplitude *V*′_*w*_ and also reduce the pulse width *t*′_*p*_ Equation (4) to further decrease the energy consumption per setting process (Figure 6).

In order to optimize the energy efficiency of BFO-based artificial synapses, we have applied a large writing pulse amplitude of 23.0 V to compensate the short pulse width of 50 ns. The corresponding energy consumption amounts to 4.7 pJ. The LRS reading current and HRS reading current at 2.0 V amount to 980 and 64 nA, respectively. The theoretical maximum normalized current ranges from 93.5 to 0% and from 0 to 93.5% in both curves Equation (2) and (3).

In Table 2 (Kandel and Schwartz, 1985; Jo et al., 2010; Chang et al., 2011; Yu et al., 2011; Kuzum et al., 2012; Wu et al., 2012) different memristor-based artificial synapses are listed and compared with respect to their energy consumption per (re)setting process. The TiN/Ti/AlO_x_/TiN/Ti memristor (Wu et al., 2012) shows the smallest energy consumption of 1.5 pJ per SET pulse. It is expected that to a certain extent the energy consumption can be further reduced by further reducing the electrode area size A. However, one has to consider that BFO is a polycrystalline thin film and that only 1–0.1% of the crystallites below the top electrode of the polycrystalline BFO are switched in single pairing STDP.

## Discussion

### Configurability

In this work single pairing STDP in BFO-based artificial synapses has been demonstrated for emulating the functionality and the plasticity of biological synapses. The waveform-defined plasticity of BFO memristors in addition to their multilevel memristive programming capability enables easy control of the STDP time windows, as evidenced by the three orders of magnitude timescale configurability shown in this paper. While there has been a lot of simulation work on this topic, the number of devices where STDP or variations have actually been implemented and measured is still fairly small (Jo et al., 2010; Alibart et al., 2012). Among those, our highly-configurable, finely grained learning curves are unique, other implementations exhibit statistical variations (Jo et al., 2010), can only assume a few discrete levels (Alibart et al., 2012) or the learning windows are device-inherent, i.e., cannot be adjusted (Ohno et al., 2011). We expect that for BFO-based artificial synapses at least 32/64 levels are possible in a power efficient manner. In addition, the wide range of timescales possible in BFO-based synapses enables e.g., a timebase-tunable system that could learn a classification offline in an accelerated manner, while still able to interact with real-time sensors before or after this learning.

As mentioned in the introduction, BFO-based artificial synapses can be used for conventional STDP experiments, where only multiple spike pairings exhibit significant weight change, as well as in the mode used in this paper, where a single pairing already induces a significant weight change. By changing the voltage of the pre- and post-synaptic pulses, any point in between these two extremes can also be chosen, again showing the excellent configurability of BFO-based artificial synapses. However, the versatility of BFO memristors comes at the price that in contrast to e.g., phase-change materials, BFO is not easily integrated on top of CMOS (Shuai et al., 2013).

### Energy consumption

In Table 2, we have shown an energy consumption of *E* = 4.7 pJ in a BFO-based artificial synapse with electrode size of 4.52E4 μm^2^. While this is still three orders of magnitude above the energy consumption of biological synapses, it is one of the lowest reported so far for other artificial synapses. Compared to neuromorphic approaches, all memristive approaches are several orders of magnitude better (Azghadi et al., 2014). In terms of absolute area, the BFO memristor is comparable to some neuromorphic implementations (Hasler and Marr, 2013; Noack et al., 2015), but not competitive with memristor crossbar devices, as we are employing a single device test structure that has a large contact size for reasons of convenience. However, BFO device scaling is well established, thus we can aggressively scale the size of the top electrode to 10 μm^2^ and the thickness of the BFO to 100 nm (Jin et al., 2014). For BFO with larger electrode area size, the current scales linearly with area size. For smaller electrode area size we would expect that the current scales with the number of BFO crystallites below the electrode. And in the limit case of nanoscale electrodes, the smallest possible current should be the current through single BFO crystallites.

### Retention of weight change

We have investigated the retention of memristance weight change across time. As Figure 5A shows, the basic shape of the STDP curves is preserved across time. Figure 5B illustrates that even after memory consolidation, we retain a graded weight, i.e., a unimodal weight distribution. Our synapse does not collapse in either a potentiated or depressed (bimodal) distribution as predicted in some synaptic models (Fusi et al., 2000; Clopath et al., 2008). In memristive literature, there is usually no investigation of these phenomena, the weight change is taken at some unspecified time after induction and then assumed to be non-volatile. Only very few articles have investigated the actual non-volatility/weight retention across time and shown that the assumption of a non-volatile change is not necessarily valid (Chang et al., 2011). Thus, compared to other reports, this article gives a neuromorphic designer a clear guide on how to utilize the memristive synapses for long-term storage.

Interestingly, this investigation of memory consolidation is also somewhat missing in the original biological measurements. Usually, data on the weight evolution ca. 30–60 min after induction is provided, but only on single example pairing experiments. These data points show various behaviors, from unchanged weights after initial weight induction (Froemke and Dan, 2002) to increases of weight change across time (Bi and Poo, 1998), decreases across time (Markram et al., 1997) or slow oscillations around the initial potentiated/depressed weight value (Sjöström et al., 2001). However, it is unclear how the overall STDP window consolidates over time. Thus, measuring the evolution of an STDP curve across time after induction at biological synapses similar to our investigation on memristive synapses may actually be a quite interesting scientific question.

## Summary and outlook

In this work we have investigated a wide range of timescale configurability, ranging from 25 ms to 125 μs. Also, we have investigated power consumption figures and have shown that it is possible to decrease the switching pulse width and to reduce the power consumption during a single STDP writing process on BFO-based artificial synapses to only 4.5 pJ. Furthermore, the increased programming voltage also shortens the total pairing spike time, and enables to move from the standard biology-like 60–80 spike pairing STDP experiment to a single pairing STDP experiment with the same weight/memristance change.

One important advantage of single STDP in comparison to 60–80 spike STDP is that both pre- and post-synaptic waveform are causal, i.e., they start only at the pre- respectively post-synaptic pulse. This is in contrast to most currently proposed waveforms for memristive learning, where the waveforms have to start well in advance of the actual pulse (Zamarreño-Ramos et al., 2011), which requires pre-knowledge of a pulse occurrence. Especially, in an unsupervised learning context with self-driven neuron spiking, this pre-knowledge is simply not existent.

In a wider neuroscience context, waveform defined plasticity as shown here could be seen as a general computational principle, i.e., synapses are not likely to measure time differences as in native forms of STDP rules, they are more likely to react to local static (Ngezahayo et al., 2000) and dynamic (Dudek and Bear, 1992) state variables. In the future some interesting predictions could be derived from that, e.g., STDP time constants that are linked to synaptic conductance changes or to the membrane time constant (Pfister et al., 2006; Mayr and Partzsch, 2010). These predictions could be easily verified experimentally.

### Conflict of interest statement

The authors declare that the research was conducted in the absence of any commercial or financial relationships that could be construed as a potential conflict of interest.
